# Impact of Robotic Assistance on Precision of Vitreoretinal Surgical Procedures

**DOI:** 10.1371/journal.pone.0054116

**Published:** 2013-01-15

**Authors:** Yasuo Noda, Yoshiki Ida, Shinichi Tanaka, Taku Toyama, Murilo Felix Roggia, Yasuhiro Tamaki, Naohiko Sugita, Mamoru Mitsuishi, Takashi Ueta

**Affiliations:** 1 Department of Ophthalmology, Graduate School of Medicine and Faculty of Medicine, The University of Tokyo, Bunkyo-ku, Tokyo, Japan; 2 School of Engineering, The University of Tokyo, Bunkyo-ku, Tokyo, Japan; Medical University Graz, Austria

## Abstract

**Purpose:**

To elucidate the merits of robotic application for vitreoretinal maneuver in comparison to conventional manual performance using an *in-vitro* eye model constructed for the present study.

**Methods:**

Capability to accurately approach the target on the fundus, to stabilize the manipulator tip just above the fundus, and to perceive the contact of the manipulator tip with the fundus were tested. The accuracies were compared between the robotic and manual control, as well as between ophthalmologists and engineering students.

**Results:**

In case of manual control, ophthalmologists were superior to engineering students in all the 3 test procedures. Robotic assistance significantly improved accuracy of all the test procedures performed by engineering students. For the ophthalmologists including a specialist of vitreoretinal surgery, robotic assistance enhanced the accuracy in the stabilization of manipulator tip (from 90.9 µm to 14.9 µm, P = 0.0006) and the perception of contact with the fundus (from 20.0 mN to 7.84 mN, P = 0.046), while robotic assistance did not improve pointing accuracy.

**Conclusions:**

It was confirmed that telerobotic assistance has a potential to significantly improve precision in vitreoretinal procedures in both experienced and inexperienced hands.

## Introduction

Recently robot-assisted surgery has broadened its application and has been introduced into the surgical theaters worldwide [1]–[4]. Advantages of robot-assisted surgery include improved dexterity and accuracy, steep learning curve, and telesurgery. Currently the da Vinci Surgical System (Intuitive Surgical Inc.) has been the major robotic system, although it can not be introduced into the intraocular microsurgery due to its large size [5]. Among the intraocular surgeries, sophisticated vitreoretinal procedures such as the peeling of the internal limiting membrane (ILM) or retinal vessel cannulation require the highest level of dexterity and accuracy, and desired positioning accuracy was considered approximately 10 µm [6] while the average amplitude of hand tremors is considered approximately 100 µm [7], which indicates that vitreoretinal surgery can be a good target of application for robotic surgery. In fact, enforcing the precision of vitreoretinal surgery by telerobotic system has been pursued [5], [8]–[13]. At first, removal of 0.015 inch diameter particle from a simulated eyeball was reported in 1997 [9]. In 2009 we reported a prototype of telerobotic system that could perform the creation of posterior vitreous detachment, retinal vessel sheathotomy and retinal vessel cannulation [10].

According to these previous studies, the potential of telerobotic systems have been shown through demonstrations of vitreoretinal surgical tasks, however, on what kind of aspects the robotic assistance would improve the quality of vitreoretinal surgical procedures has been remained unclear. For example, how the robotic assistance can affect the different kinetic aspects of surgical procedures or how does it influence the surgeons with different volume of surgical experience? In the current study we constructed an eye model for vitreoretinal surgical tasks and addressed these issues.

## Materials and Methods

### Construction of an *in-vitro* Eye Model Simulating the Environments of Vitreoretinal Surgery

**Figure 1:**
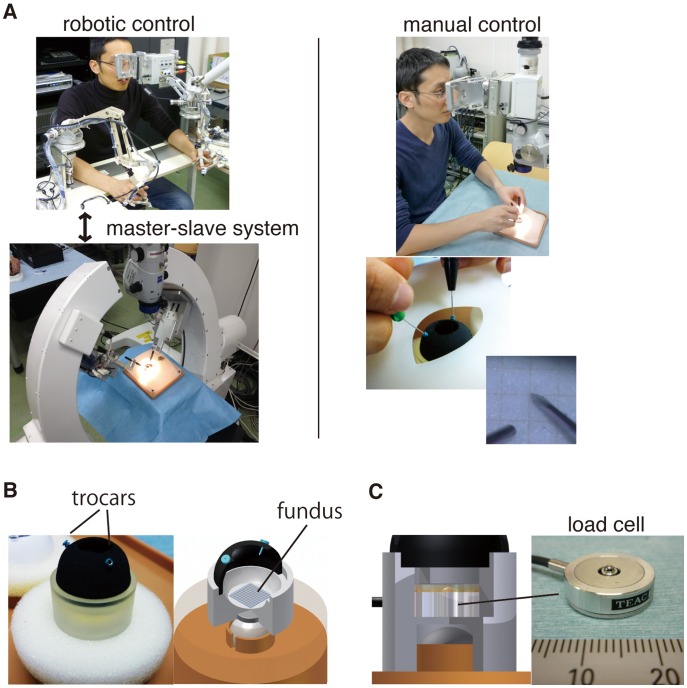
Robotic/manual control of a vitreoretinal surgical instrument and an *in-vitro* eye model constructed for the present study. (A) Test procedures were conducted manually and by telerobotic system using the same visual system. A 25G v-lance was introduced into the eye through trocar cannula. (B) A piece of graph paper was attached on the fundus that provided targeting points for the test procedures. The eye model was loosely fixed by a magnet to mimic the eye movement during surgery. (C) In the test procedure 3 to measure the foce applied on the fundus, a load cell was placed beneath the graph paper of the fundus.

An overview of the *in-vitro* eye model constructed for the current study is shown in Figure 1. The *in-vitro* eye model of 24 mm diameter had 8 mm diameter opening on its top as mydriatic pupil, and was made of 1.5mm-thick rubber. Because the elasticity (i.e., Young’s modulus) of the human sclera is reportedly 2.7±1.4 MPa [14], the anterior part of the model was made to approximate the elasticity. Commercially available 25G trocar cannula (Alcon, Inc.) was placed 3 mm behind the edge of the opening on the top as performed in vitreoretinal surgery. The posterior, or bottom, part of the eye model was made of plastic material by rapid prototyping. The test procedures were performed on the bottom or fundus inside the eye model. The eye model was loosely fixed with a magnet and sponge to mimic the movement of the eye by the force applied through the microsurgical instruments at the trocars. The basement of the eye model including the magnet and sponge, as well as the face cover over the eye model was from commercially available KITARO WetLab [15].

### Test Environment

Details of the robotic system used in the current study were explained elsewhere [10], [13]. Briefly, the master-slave robotic system [13] was used in the present study. Each slave manipulator had 5 degrees of freedom that enabled surgical instrument’s movement in the intraocular space, rotation around the instrument and grasping. The slave manipulators had a remote center of motion that was mechanically guaranteed, and the remote center of motion was designed to correspond to the inserting point of a trocar cannula. During an operation, the hand motion of a surgeon is measured by the master manipulators and scaled down by 1/40 and then transmitted to the slave manipulators performing the operation in real time in order to increase the accuracy. Microscopic vision at the operation site was transmitted to the master site with 3D vision. The examinees introduced a 25 G V-lance knife (Alcon, Inc.) and a light probe into the eye through 25G trocar cannulae manually or by robotic control. For both manual and robotic control, examinees used the same 3D viewing system to perform the test procedures. To compare the accuracy by experienced and inexperienced hands, 4 ophthalmologists including a specialist of vitreoretinal surgery and 5 engineering students were enrolled.

### Test Procedures

To examine different aspects of vitreoretinal surgical tasks 3 test procedures were evaluated in the present study. Firstly, aiming accuracy represents how accurately the tip of the tool can be controlled to approach the designated targets on the fundus. Secondly, positioning stability was used to determine how the tip of the tool can be stabilized at the same position. This skill is important when drugs are being infused into the retinal vessel after retinal vessel cannulation, or when ILM forceps is positioned on the retinal surface and grasp the initial ILM flap. Thirdly, fine perception of contacting with the fundus is critically important to preserve intact retina while performing surgeries on the fundus.

### Test Procedure 1; Aiming Accuracy

In this test procedure a V-lance knife was held manually or by the slave manipulator of the robotic system. Then the V-lance was introduced into the eye model through a trocar, and an examinee was asked to touch 4 target points on the fundus (Movie S1). Firstly the examinee was asked to conduct the procedure by manual control, then using the robotic control afterward. This set of procedure was repeated 3 times. This meant that 12 aiming accuracy procedures were conducted for both robotic and manual controls of each examinee. The procedure was repeated alternately by manual and robotic controls to equalize the learning effect. The mean deviation (µm) from the target was measured based on the recorded movie. The procedure was conduced by 5 engineering students and 4 ophthalmologists including a specialist of vitreoretinal surgery.

### Test Procedure 2; Positioning Stability

In this test procedure, V-lance knife was held manually or by the slave manipulator and then inserted into the eye model through a trocar as conducted in the test procedure 1. The tip of the V-lance was positioned just above a target point and the examinees were asked to keep the position for 1 min (Movie S2). The maximum deviation (µm) from the target point during the 1 min was measured based on the recorded movie and the mean values in the same 3 procedures for each examinee was calculated. The manual and robotic procedures were alternately repeated. This test procedure was conduced by 5 engineering students and 4 ophthalmologists including a specialist of vitreoretinal surgery.

### Test Procedure 3; Perception of Contacting with the Fundus

**Figure 2:**
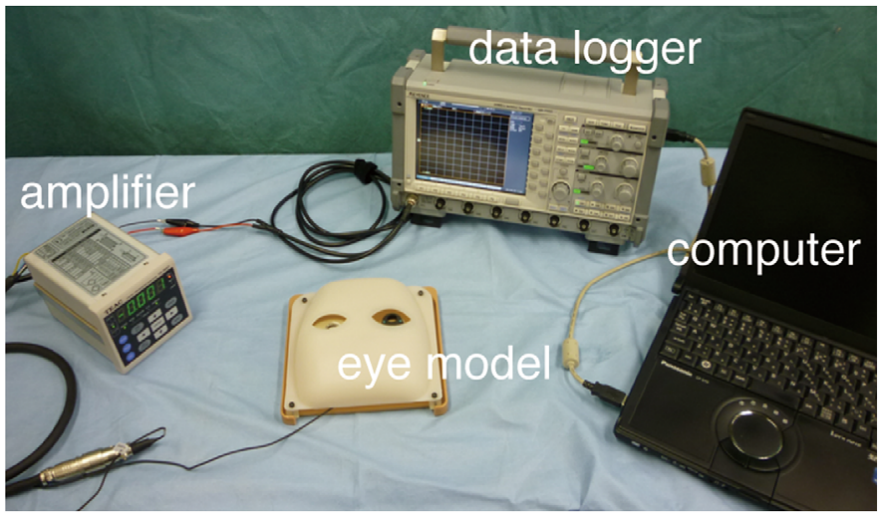
System to evaluate force applied on the fundus for test procedure 3, perception of contact with the fundus. An amplifier detected the voltage changes converted from the applied force change on the load cell. The amplifier was calibrated to measure 30 mN as 1 V change. The values of changes in voltage were shown with noise filtering in the data logger, and recorded on the computer.

In this test procedure a 25 G V-lance knife, held manually or by slave manipulator of the robotic system, was inserted through a trocar and the examinees were asked to touch the fundus of the eye model with as minimal force as possible (Movie S3). The force applied on the fundus was measured by a load cell (TC-USR-17, TEAC) beneath the graph paper (Figure 2). Furthermore, using the robotic system, when the V-lance moves 0.2 mm downward the surface of the fundus, a force of 25.7 mN was recorded. This meant that touching the fundus with 1 mN force in this eye model would result in 7.7 µm invasion beyond the surface. Based on this, force by mN was converted to distance by µm through a conversion coefficient of 7.77 for clearer understanding of the applied force in the settings of vitreoretinal surgery. An examinee contacted the fundus 4 times manually, and then 4 times by robotic control. The set of the procedure was repeated 3 times alternately by manual and robotic controls. Five engineering students and 4 ophthalmologists including a specialist of vitreoretinal surgery performed this task.

### Statistical Analysis

Statistical analyses were performed using JMP9 software (SAS). P value less than 0.05 was regarded as statistical significance. The numerical values were analyzed through 2-tailed student’s paired or unpaired *t-*test based on the conditions of the experiments.

## Results

### Aiming Accuracy

**Figure 3:**
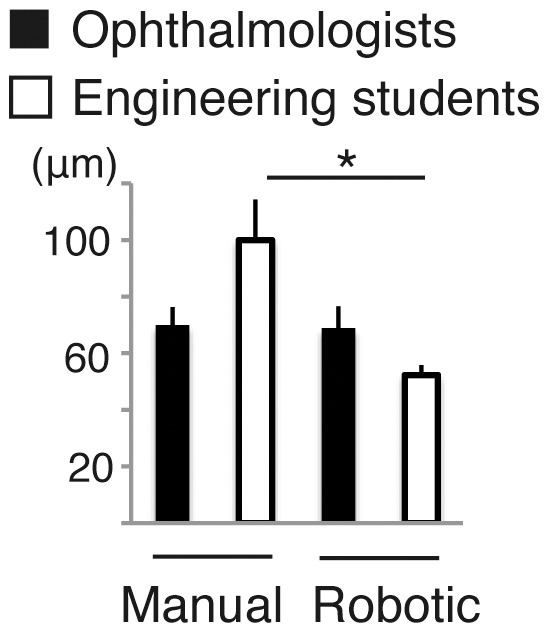
Comparison of manual and telerobotic control by ophthalmologists and engineering students in test procedure 1, aiming accuracy. (mean ± SEM; n = 4−5 per group; *P<0.05).

Result on aiming accuracy was shown in Figure 3. Manually, the mean aiming accuracy by doctors (69.9 µm) was superior to that by engineering students (100.0 µm), although the difference was not statistically significant (P = 0.176, by unpaired student’s *t*-test) probably due to the small sample size. By use of robotic assistance, the accuracy was significantly improved when used by the students (52.3 µm, P = 0.031, by paired *t*-test). Aiming accuracy did not change in doctors with or without the robotic assistance.

### Positioning Stability

**Figure 4:**
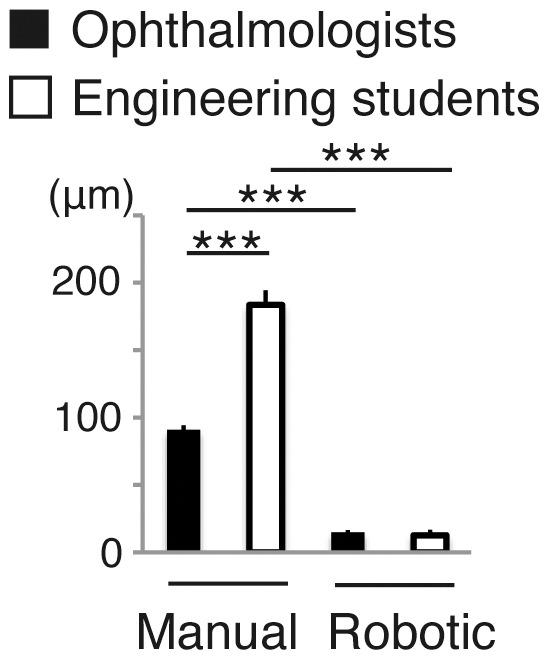
Comparison of manual and telerobotic control by ophthalmologists and engineering students in test procedure 2, positioning stability. (mean ± SEM; n = 4−5 per group; ***P<0.001).

Result on positioning stability was shown in Figure 4. Manually, the difference in the mean positioning stability was significant (P = 0.0002, by unpaired student’s *t*-test) between doctors (90.9 µm) and students (183.5 µm). By the use of the robotic system, positioning accuracy was profoundly improved both in doctors (14.9 µm, P = 0.0006, by paired *t*-test) and in students (12.6 µm, P = 0.0002, by paired *t-*test), and the stability in students was as good as that in doctors with robotic assistance.

### Perception of Contacting with the Fundus

**Figure 5:**
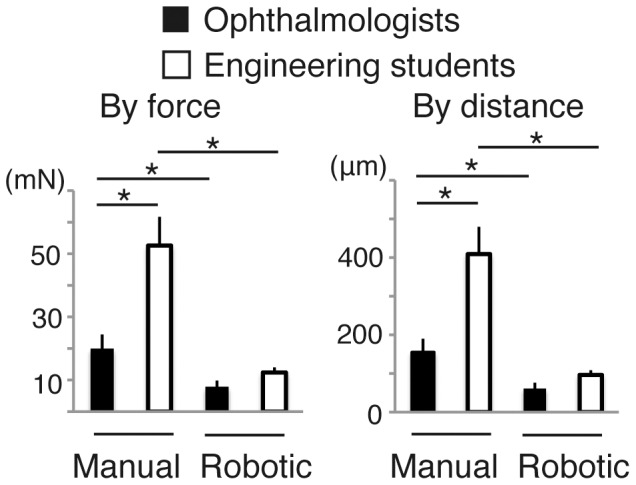
Comparison of manual and telerobotic control by ophthalmologists and engineering students in test procedure 3, fine perception of contact with the fundus. (mean ± SEM; n = 4−5 per group; *P<0.05).

Result on the fine contact with the fundus was shown in Figure 5. Manually, the mean force to touch the fundus delicately was 20.0 mN in the doctors and 52.6 mN in the students (P = 0.040, by unpaired student’s *t*-test). By employing the robotic assistance, the delicacy was again profoundly improved both in doctors (7.84 mN, P = 0.046, by paired *t-*test) and in students (12.4 mN, P = 0.012, by paired *t-*test). A vitreoretinal surgery specialist was especially delicate to touch the fundus (11.3 mN manually and 3.97 mN by the robot). Converted distance data was also presented in figure 5.

## Discussion

Vitreoretinal surgery is performed using instruments introduced into the eye through trocars set at the designated place, and the instruments have to be controlled in a pivot movement around the trocars. It has not been clear yet how a robotic system would help in such a peculiar and well-designed environment of vitreoretinal surgery. Our results indicate that a robotic system for vitreoretinal surgery improve several kinetic aspects of vitreoretinal surgical procedures by both experienced and inexperienced hands. The significant difference in accuracy between ophthalmologists and students was found in the positioning stability and perception to touch the fundus. However, in these tasks the robotic assistance dramatically improved the quality of performances, and ophthalmologists and students conducted the tasks in equal and higher quality.

How robotic surgery affects surgeons with different volume of experience has been discussed in other fields of surgery. For example, in a setup of laparoscopic surgery [16], a robotic system helped novices more than experts. Also the learning curve could be improved and achieved faster using a robotic assistance [17]. In the present study, the robotic assistance improves performances of not only novices but also experts in 2 of the 3 tasks, i.e., positioning stability and perception of contacting with the fundus. In the task of the aiming accuracy, there was a significant benefit of using robotic system for engineering students, but not for ophthalmologists. Our results suggest that the impact of robotic assistance could be different depending on the kinetic aspects of procedures as well as volume of experience.

For the test procedure of positioning stability, we have conducted the same *in-vitro* test in our previous studies, but without the eye model [11]. Without the eye model the maximum deviation from the target point was more than 200 µm. The deviation was much smaller in the present study with the eye model, indicating that trocar cannula confers additional stability for the instruments during vitreoretinal surgery.

In contrast to many other surgeries, manipulation on the fundus is below the lower threshold of human tactile perception [18], [19] which means that the perception of touching the funds is through visual information. In the present study largest difference in the quality of performance between the ophthalmologists and students were found in the test procedure of perception of contact with the fundus. Especially, the specialist of vitreoretinal surgery was profoundly superior in this aspect. He perceived the touch on the fundus with 11.3 mN and 3.97 mN in manual control and robotic assistance, respectively. In contrast, other ophthalmologists perceived with around 20−25 mN and 10 mN in manual control and robotic assistance, respectively, and engineering student perceived with more than 50 mN and 10 mN in manual control and robotic assistance, respectively. These results indicate that fine perception of contact with the fundus depends on surgical experience, and robotic assistance could be especially useful in this aspect of vitreoretinal procedures.

Other than the master-slave robotic system that we are using, several other engineering concepts to assist vitreoretinal surgery have been pursued. Mitchell et al. have developed a steady-hand system [20]. This system is a handheld device where the surgeons and system share control of the instrument with force sensors. The force information at the tip of the instrument is used to provide smooth, tremor-free, and precise positional control and force scaling. Additionally, microcannulation of an 80-µm blood vessel was successfully demonstrated using chicken embryos.

Iordachita et al. have developed a microforce sensor to detect small contact forces between instruments and tissues [21]. They integrated steady-hand systems and a microforce sensor to perform highly accurate and safe maneuvering of surgical instruments using sensor feedback [22]. Choi et al. have developed the Micron system [23]. This handheld system is capable of detecting the movement of a surgeon’s hand to distinguish between desired and undesired motions, and the system cancels the undesired motions using a piezo actuator. They successfully reduced the amplitude of hand tremors from 91 to 60 µm peak–peak, and thus, highly accurate and stable positioning of the tool and reduced hand tremors have been achieved, although the skill of the surgeon influenced the positioning accuracy. Robot-assisted vitreoretinal surgery is still at an immature stage of its development, however, these strategies with different engineering concepts can be fruitful in the future.

One of the limitations for the current study was that because the test procedures needed to be operated in a simulated eye-model, the operating conditions did not perfectly match the actual situations of vitreoretinal surgery in human patients. For example, although our eye model was loosely fixed to reproduce the eye movement during surgery, we could not reproduce the head movement of the patients that sometimes occurs during vitreoretinal surgery which is performed under local anesthesia. In addition, when contacting the ILM in human patients, subtle visual information such as distortion of nerve fiber layer or retinal surface by the contact of the instrument tip is important, though it was difficult to be reproduced in the rigid fundus of the eye model used in the present study. This is considered why the measured values of force or distance in the test procedure of perception of contact with the funds were relatively large. However, comparison of manual and robotic performance under the same conditions was more important for the purpose of the present study.

In conclusion, through the *in-vitro* evaluation of robot-assisted vitreoretinal surgical procedures compared to manual procedures, benefits of robotic assistance for the sophisticated vitreoretinal surgeries was confirmed.

## Supporting Information

Movie S1 (WMV)

Movie S2 (WMV)

Movie S3 (WMV)
